# Measurement of the D/H, ^18^O/^16^O, and ^17^O/^16^O Isotope Ratios in Water by Laser Absorption Spectroscopy at 2.73 μm

**DOI:** 10.3390/s140509027

**Published:** 2014-05-21

**Authors:** Tao Wu, Weidong Chen, Eric Fertein, Pascal Masselin, Xiaoming Gao, Weijun Zhang, Yingjian Wang, Johannes Koeth, Daniela Brückner, Xingdao He

**Affiliations:** 1 Key Laboratory of Nondestructive Test (Ministry of Education), Nanchang Hangkong University, Nanchang 330063, China; E-Mails: wutccnu@nchu.edu.cn (T.W.); xdhe@126.com (X.H.); 2 Laboratoire de Physicochimie de l'Atmosphère, Université du Littoral Côte d'Opale, 189A, Av. Maurice Schumann, 59140 Dunkerque, France; E-Mails: eric.fertein@univ-littoral.fr (E.F.); pascal.masselin@univ-littoral.fr (P.M.); 3 Anhui Institute of Optics & Fine Mechanics, Chinese Academy of Sciences, Hefei 230031, China; E-Mails: xmgao@aiofm.ac.cn (X.G.); wjzhang@aiofm.ac.cn (W.Z.); wyj@aiofm.ac.cn (Y.W.); 4 Nanoplus Nanosystems and Technologies GmbH, Oberer Kirschberg 4, 97218 Gerbrunn, Germany; E-Mails: johannes.koeth@nanoplus.com (J.K.); daniela.brueckner@nanoplus.com (D.B.)

**Keywords:** isotope ratios, water, Kalman filtering, laser absorption spectroscopy

## Abstract

A compact isotope ratio laser spectrometry (IRLS) instrument was developed for simultaneous measurements of the D/H, ^18^O/^16^O and ^17^O/^16^O isotope ratios in water by laser absorption spectroscopy at 2.73 μm. Special attention is paid to the spectral data processing and implementation of a Kalman adaptive filtering to improve the measurement precision. Reduction of up to 3-fold in standard deviation in isotope ratio determination was obtained by the use of a Fourier filtering to remove undulation structure from spectrum baseline. Application of Kalman filtering enables isotope ratio measurement at 1 s time intervals with a precision (<1‰) better than that obtained by conventional 30 s averaging, while maintaining a fast system response. The implementation of the filter is described in detail and its effects on the accuracy and the precision of the isotope ratio measurements are investigated.

## 1. Introduction

The stable isotopes of water (in the vapor or liquid phase) are powerful tracers for the study of the hydrological cycle, climate change, ecological process and paleoclimatic archives (see, e.g., [1–4]), as well as for biomedicine [5]. Analysis of the stable isotope abundance has been the domain of stable isotope ratio mass spectrometry (IRMS), which generally achieves a very high precision, but IRMS is incompatible with condensable gases like water. In the case of water isotopologue analysis, reduction of water to H_2_ and equilibration with CO_2_ is commonly used for the determination of the ^2^H (or D) and ^18^O isotope ratios [6,7]. In addition, as the ^17^O^12^C^16^O and ^16^O^13^C^16^O molecules appear in the same mass channel, accurate and direct determination of the isotope ratio H_2_^17^O/H_2_^16^O is practically impossible. This ratio is usually inferred from the natural relation between the ^17^O and ^18^O abundance ratios [8] or determined on O_2_ after fluorination of the water sample [9]. No reliable high precision mass spectrometric method is available for direct isotopic analysis of water. Moreover, since the equipment is quite bulky it cannot be used in the field and is generally incapable of performing real-time measurements.

Measurements of the stable isotope ratio by optical spectroscopy, in particular by laser absorption spectroscopy (LAS), have attracted a growing interest in recent years [10,11]. Laser spectroscopy provides an excellent opportunity to perform *in situ* real-time continuous measurements without requiring chemical conversion. Nowadays isotope ratio laser spectrometry (IRLS) becomes a serious competitor to IRMS. Water isotope spectrometers operating in the near infrared around 1.4 μm [12–14], in the mid infrared at 2.7 μm [15,16] and 6.7 μm [17,18] have been developed. Cavity enhanced spectroscopy approaches have been implemented for achieving high sensitivity and high precision [18–20]. High precision IRLS instruments are now commercially available from LGR Inc. (Mountain View, CA, USA) (using off-axis Integrated Cavity Output Spectroscopy, known as OA-ICOS) [21–23] and from Picarro Inc. (Santa Clara, CA, USA) (using Cavity Ring Down Spectroscopy—CRDS) [24–27].

In this paper, we report on the development of a compact IRLS instrument for simultaneous real-time measurements of the D/H, ^18^O/^16^O and ^17^O/^16^O isotope ratios in water by laser absorption spectroscopy at 2.73 μm. The 2.7 μm fundamental stretching mode is about one order of magnitude stronger than the band at 1.4 μm, and even the bending mode near 6.7 μm is not much stronger than that the 2.7 μm band. More important is that the selected absorption lines at 2.73 μm for measurements of water isotopologues (H_2_^18^O, H_2_^16^O, H_2_^17^O and HDO) may have relatively similar line strengths and similar ground state energies (Table 1). Recently available, commercial 2.73 μm distributed feed-back (DFB) lasers offer the following advantages compared to a color center laser previously used for water isotopologue measurement [15]: single mode and single frequency emission at room temperature, high electronic bandwidth (as high as kilohertz repetition rates), in combination with compactness (mounted inside a TO-8 package) and cost-effectiveness [16].

In the present work, special attention is paid to the spectral data processing by use of digital filtering techniques to improve the measurement precision. Oscillatory structure on spectrum baseline affecting the measurement precision was analyzed. Fourier analysis of spectral residuals resulting from a fit was performed, which enabled the identification and filtering of the noise in spectral signals with the help of Fourier filter. The paper also provides a detailed description of Kalman filtering of the data, as recently introduced by us to the field of isotope ratio measurements [28]. We demonstrate that a faster temporal response (1 s) can be achieved by Kalman filtering with a precision better than that obtained by conventional 30 s averaging. Effects of Kalman filtering on the accuracy and the precision of the isotope ratio measurements are also studied in the present work.

## 2. Experimental Consideration

### 2.1. Isotopic Composition Analysis by Laser Absorption Spectroscopy

According to the Beer-Lambert law of linear absorption, the absorbance *A*(*v*) can be related to the transmitted light *I*(*v*) and incident intensity *I*_0_(*v*) intensities:

(1) $$A(v)=\ln\;(I_{0}(v))/I(v\text{)}=C\sigma (v)L$$

where *C* is the number density of absorbing species (in mol/cm^3^), *σ*(*v*) is the frequency-dependent absorption cross section (in cm^2^/mol), and *L* is the optical absorption path length (in cm). The integrated absorbance *A_I_* (in cm^−1^) can be written as:

(2) $$A_{I}=\int A(v)dv=\int \ln(I_{0}(v))/I(v))dv=CL\int \sigma (v)dv=\text{CLS}(T)/n$$

with *S*(*T*) the temperature-dependent molecular line absorption intensity in cm^−1^/(mol·cm^−2^) and *n* the fractional isotope abundance; both can be found in the HITRAN database [29]. The dependence of the absorption line intensity *S*(*T*) on the temperature T can be described as follows [13]:

(3) $$S(T)=S(T_{0}){\left(\frac{T_{0}}{T}\right)}^{3/2}\exp\left[-\frac{hcE_{0}}{k}\left(\frac{1}{T}-\frac{1}{T_{0}}\right)\right]$$

with T_0_ = 296 K, E_0_ the lower level energy expressed in cm^−1^, *h* the Planck constant (J s), *c* the speed of light in vacuum (cm/s) and *k* the Boltzmann constant (J/K).

The isotope ratio can thus be determined from the ratio of the integrated areas *A_I_* and the absorption line intensities S of the major and minor isotopic components:

(4) $$R=[C^{x}]/[C^{a}]=\frac{A_{I}^{x}}{A_{I}^{a}}\times \frac{S^{a}/n^{a}}{S^{x}/n^{x}}$$

where *x* refers to the rare isotopic species (H_2_^17^O, H_2_^18^O, or HDO), *a* represents the abundant isotopic component (H_2_^16^O), and *R* represents the ratio of the rare to the abundant isotopologues abundances.

The relative deviation of the isotope ratio in water with respect to the international standard reference known as Vienna Standard Mean Ocean Water (VSMOW), is expressed in terms of the δ-value:

(5) δ(‰)=R−sRVSMOWRVSMOW×1000

where, by international convention, *R_vsmow_* takes on the values: *R_vsmow_* = 0.0020052 for ^18^O, 0.0003799 for ^17^O and 0.00015576 for ^2^H [30]. It is noted, however, that substitution of Equation (4) into Equation (5) results in an expression for the delta-value in which the ratios of the line strengths cancel, provided that the sample and reference spectra are recorded at the same temperature (see, e.g., Reference [10]). In practice, the laser spectrometer is calibrated against working reference standards with isotopic ratios that are well known on the international two-point scale (determined by the consensus values for VSMOW and Standard Light Antarctic Precipitation—SLAP), recommended by the IAEA [30]. A few years ago, the International Atomic Energy Agency (IAEA) produced the new reference material VSMOW2 to replace the exhausted reference material VSMOW. Repeated measurements of VSMOW and VSMOW2 by a number of international laboratories have shown that they have indistinguishable δ^18^O, δ^17^O and δ^2^H values, and thus indistinguishable *R_vsmow_* for ^18^O, ^17^O and ^2^H [31].

In the present work, the laser instrument was calibrated against the working standards GS-49 (δ^18^O = 0.39‰, δ^17^O = 0.21‰, and δ^2^H = 1.7‰, with respect to VSMOW, determined by repeated IRMS analyses at the Center for Isotope Research of the University of Groningen). The accuracy of the laser instrument was evaluated by measurement of another working standard GS-42 with different isotopic composition (δ^18^O = −24.62‰, δ^17^O = −13.1‰, and δ^2^H = −187.7‰). Bottled water (Vittel, France) was used as unknown sample material.

### 2.2. Selection of the Absorption Lines

Selection of suitable absorption lines for water isotopic ratio measurements is one of the most important aspects in instrumental design since the choice of absorption lines has a direct impact on the instrumental performance in terms of measurement sensitivity, precision and selectivity.

Measurements of the isotopic ratios δ^18^O, δ^17^O, δ^2^H of the stable isotopologues of water require probing of absorption lines of the four isotopologues H_2_^18^O, H_2_^16^O, H_2_^17^O and HDO within the laser tuning range. It is very desirable that the used absorption lines exhibit similar absorption depths at natural abundance with an absorption intensity as large as possible (for high sensitivity and high precision measurements), are free from interference from the same or other species (high selectivity consideration), and have similar ground state energy (in order to minimize the effects of temperature-dependent line intensities). The spectral region near 2.73 μm, covered by recently commercially available DFB lasers, meets these requirements to a high degree. Parameters of the four molecular lines selected in the present work are summarized in Table 1. Parameters of the previously used lines at 1.4 μm and 6.7 μm are also given for comparison.

The line absorption strength depends on the population of the ground state level and this population in turn depends on the temperature. Temperature drift during measurements may introduce a systematic error in the isotope ratio determination. The temperature coefficients defined as ΔS/S(T_0_) for the selected lines are listed in Table 1. A thermal drift of 1 K would lead to a relative variation in the line strength of +1.5‰, +4.6‰, −1.4‰, −3.4‰ for the selected lines of H^18^OH, H^16^OH, H^17^OH and H^16^OD respectively. Therefore, realization of a temperature stability better than 0.1 K is essential for high precision measurements.

## 3. Experimental description

### 3.1. Experimental Set-Up

**Figure 1.**
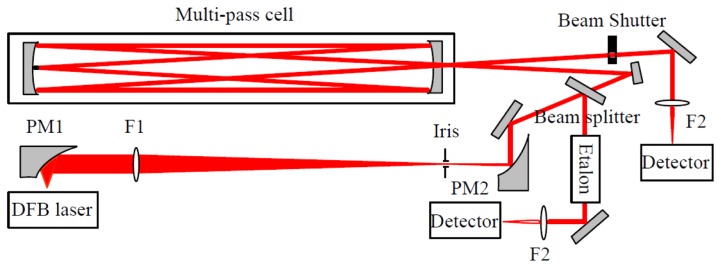
Optical layout. PM1 and PM2: parabolic mirrors with effective focal length of 25 mm and 50 mm, respectively; F1 and F2: lenses of focal length of 200 mm and 50 mm, respectively.

The experimental set-up, mounted on a 50 × 70 cm^2^ optical breadboard, is depicted in Figure 1. The laser source was a room temperature single mode DFB diode laser operating at 2.73 μm (Nanoplus GmbH, Gerbrunn, Germany). It is tunable from 2729 to 2732 nm with an output power of 2 mW. A Thorlabs (Newton, NJ, USA) ITC 502 diode laser controller provided the laser temperature control and laser drive current. The diverging laser beam was collected by an off-axis parabolic mirror PM1 with an effective focal length (EFL) of 25 mm. The laser beam was then transformed into a quasi-parallel beam with a diameter of ∼4 mm by a combination of an antireflection coated CaF_2_ lens F1 (f = 200 mm) and an off-axis parabolic mirror PM2 (EFL = 50 mm).

The collimated beam was then divided into two parts. The first part (∼8%) was reflected by a beam splitter (CaF_2_) and directed to a homemade Fabry-Perot etalon for spectral metrology. The frequency scale was linearized by means of the interference fringes produced by the etalon consisting of two air-separated uncoated CaF_2_ plates with a free spectral range (FSR) of ∼0.0283 cm^−1^. Positions of the H_2_O vapor absorption lines provided by the HITRAN database [29] were used as absolute frequency reference for laser frequency calibration. The main part of the laser beam was coupled into a Herriott cell with an absorption path of 20 m in a 36-pass configuration. The emerging absorption signal was focused with a 50 mm CaF_2_ lens F2 onto a LN_2_ cooled HgCdTe detector (J15D22-M204-S01M-60). A home-built bridge circuit was employed to realize DC coupling of the HgCdTe detector to a low noise preamplifier (Model 5113, EG&G, Albuquerque, NM, USA).

The signal background (corresponding to “laser off”), determined by the dark current level of the detector, was found to be fluctuating by about 1% over a 1 h time interval. This probably was due to variation of the detector temperature or its DC power supply. In order to correctly retrieve absorption spectra, a beam shutter (Thorlabs, SH05) was placed before the detector for background level acquisition at the beginning of each absorption spectral scan.

The laser frequency was periodically scanned at a rate of 10 Hz across the absorption lines of the water isotopologues H^16^OH, H^17^OH, H^18^OH, and HDO by means of a triangular wave voltage. The output signal from the preamplifier was digitized with a laptop using a 16-bit analogue/digital data acquisition card (DAQ Card-6036E, National Instruments, (Austin, TX, USA) controlled with a Lab Windows-based program.

### 3.2. Experimental Protocol

Careful attention has been paid in the present work to sample mass effects and sample memory effects that affect the determination of the isotopic ratios, as discussed in detail by, among others, Kerstel *et al.* [15] and more recently Lis *et al.* [21]. Liquid water samples of 12 μL were injected into the pre-evacuated gas cell through a silicon membrane using a syringe resulting in a water vapor pressure of ∼4.5 mbar inside the gas cell (the saturated vapor pressure is ∼42 mbar), which corresponds to a H_2_O molecule number density of 1.1 × 10^17^ mol/cm^3^ in the cell. Besides temperature fluctuations, variations in sample pressure inside the cell, resulting from sample injection by a syringe through a silicon membrane, will have a strong effect on the precision and accuracy of the measurements. Although constant volume was used for both sample and reference standard injections in our experiments, variances in the H_2_O number density in the cell may occur due to H_2_O leakage through the silicon membrane, over-tightening or under-tightening of the silicon membrane. In order to minimize the impact of this effect on the final results, only those injections resulting in a gas cell pressure within ±0.1 mbar of 4.5 mbar were accepted. Furthermore, in order to avoid sample memory effects due to the “stickiness” of water on the gas cell wall, the first three injections were discarded for each isotope ratio determination.

The temperature of the gas cell was actively controlled to 30 °C by the use of a heater band, and maintained constant within ±0.1 °C by a PID controller. The cell temperature was monitored with calibrated platinum resistors (Pt100) with an accuracy of 0.03 °C and a precision of 0.01 °C. No temperature gradient along the cell axis was observed within the measurement precision of the temperature sensors.

In our experiment, a slight drift of the laser wavelength with time has been observed. In order to minimize the effects of the instrumental drift on the determination of *A_I_*, only 10 laser scans were co-added, resulting in a raw δ-value at 1Hz averaged data acquisition rate. Further improvement in precision has been achieved through the use of Kalman filtering technique. As can be seen in the results presented in Section 5, the use of Kalman filtering approach allows minimizing the effect of laser wavelength drift since it filters the measurement data at 1 s time intervals.

## 4. Data Processing and Retrievals

**Figure 2.**
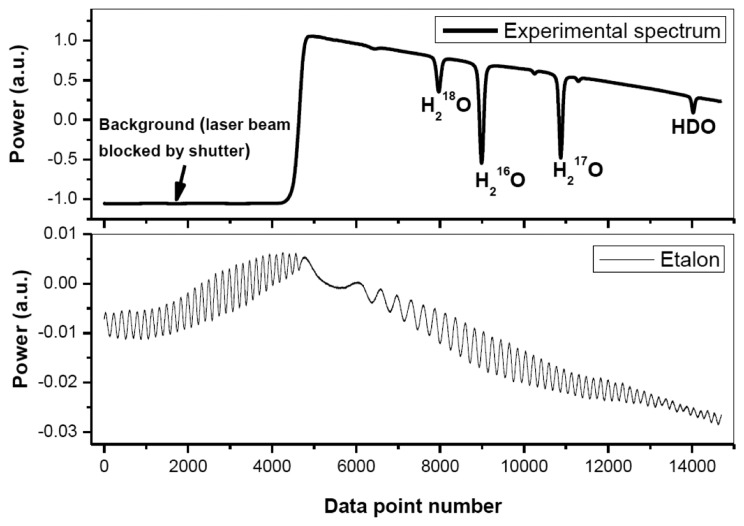
Experimental spectrum of H_2_O isotopologues around 2.73 μm. At the bottom is shown the fringe from an etalon that was used for frequency metrology in combination with the H_2_O line positions provided by the HITRAN database.

Figure 2 shows a typical experimentally recorded spectrum of H_2_O isotopologues around 2.73 μm. The spectrum, resulting from an average of 10 laser scans, was recorded at a pressure of 4.5 mbar and a temperature of 30 °C. Spectral data processing for isotopic composition determination is discussed in detail in the following subsections.

### 4.1. Spectral Fitting Algorithm

**Figure 3.**
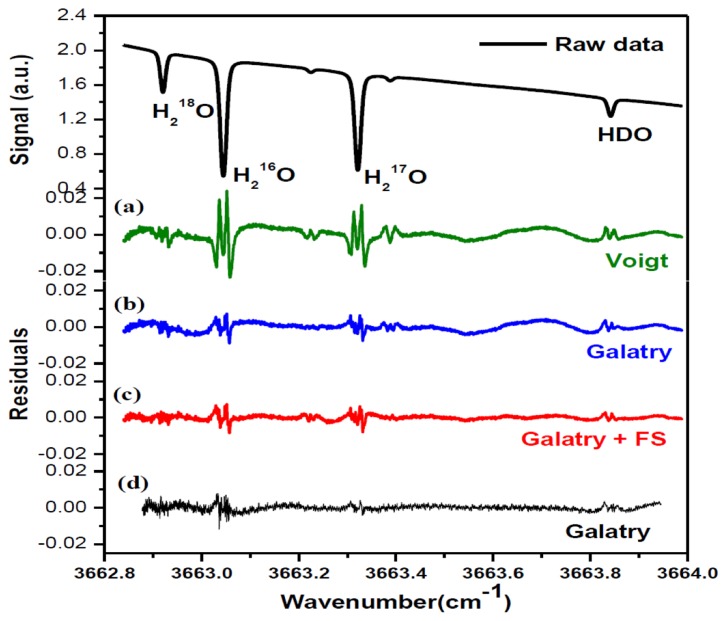
H_2_O isotopologue absorption spectrum measured in this work (raw data). Spectral data were fitted to Voigt and Galatry profiles. Residuals resulting from each fit are shown: (**a**) from Voigt fit and (**b**) from Galatry fit, respectively. (**c**) shows the fit results using Galatry profiles in combination with a Fourier series function (FS) to remove oscillation structure from the spectrum baseline. (**d**) shows a fit residual from a spectrum in the absence of an undulating structure on the baseline.

In order to determine the integrated absorbance *A_I_* (the area under the absorption line profile), absorption spectra were fitted to Voigt [32] and Galatry [33] profiles, using a Levenberg-Marquardt multi-line fitting algorithm. In the fit procedure, the baseline approximated with a 4th-order polynomial was experimentally found to well represent laser power ramp, allowing good removal of the variation of laser power in the fitted residual. Apart from the four selected lines of H^18^OH, H^16^OH, H^17^OH and H^16^OD, two H^16^OD lines at 3663.2250 cm^−1^ and 3663.3879 cm^−1^ on either side of the H^17^OH line were also taken into account in the spectral fitting program. As indicated by the fit residuals shown in Figure 3, the Galatry profile (b) fit resulted in a better residual than using the Voigt profile (a). In fact, the Voigt profile does not take into account correlations between molecular velocities and collisional processes. Corrections for velocity-changing collisions are included in the soft-collision (Galatry) model [33]. Speed dependence of the relaxation rates can be accounted for with the speed-dependent (SD) Voigt profile [34]. Both Galatry and SD-Voigt profiles are reported for recovering the experimentally observed lineshapes, and Galatry profile essentially behaves like the speed-dependent Voigt model [35]. However, Galatry profile and SD-Voigt profile are based on completely different processes. As can be seen in Figure 3c, d, relatively big residuals around absorption peaks are still evidenced even using Galatry model, which leads to a bigger uncertainty in the determination of the integrated area A_I_. In order to significantly minimize the uncertainty in the determination of the integrated area under the absorption features, a more sophisticated model could be adopted in the future (for instance, the speed-dependent Galatry profile), to take into account the narrowing due to the speed-dependence of relaxation rates as well as the averaging effect of velocity-changing collisions.

### 4.2. Suppression of Oscillation Structure on the Baseline

**Figure 4.**
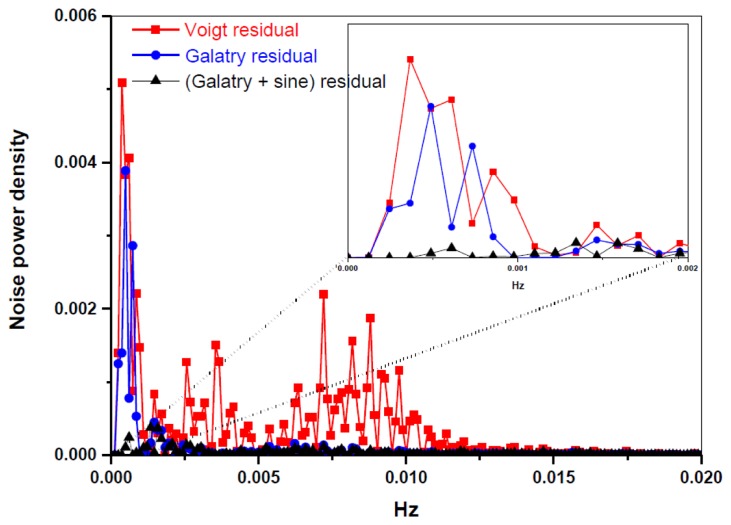
Discrete Fourier transform of the fit residuals resulting from different spectral fitting algorithms.

A periodic oscillatory structure on the baseline was observed in both the Voigt (Figure 3a) and the Galatry fit residuals (Figure 3b). This kind of sinewave-like undulation, usually assumed to be fringes resulting from optical interference, was, however, also observed with laser turned off. This means that the periodic oscillatory structure was caused by an electrical perturbation. In order to further improve the measurement precision, we applied a Fourier filtering approach [18] to suppress this oscillatory baseline structure. For this purpose, Fourier transformation of the residuals of the fit was performed in order to determine the oscillation frequencies. As can be seen in the noise power spectral density graph (Figure 4), the noise at frequencies higher than 0.001 Hz was associated with the Voigt fit residual as it has been almost completely removed in the Galatry fit. We determined that the noise peaks at frequencies lower than 0.001 Hz were associated with the baseline oscillatory structure. In order to take this undulation structure into account, the baseline was then modeled as a composition of a 4th-order polynomial and a Fourier series (FS) function of sine and cosine waves (for simulation of the sinewave-like oscillatory structure of the baseline). The transmitted power signal recorded by experiment was thus fitted to the following function:

(6) $$P=P_{0}(m)\exp(-G(m))(1+\sum \left(A_{k}\sin(\omega_{k})+B_{k}\cos(\omega_{k}))\right)$$

where m is acquired data point number, P_0_(m) is a 4th-order polynomial representing the laser power ramp, A_k_ and B_k_ are the oscillation amplitudes, and ω_k_ is the oscillation frequency determined from the Fourier transform. In our experiments, only the most prominent three frequencies have been taken into account: ω_1_= 3.662 × 10^−4^ Hz, ω_2_ = 4.882 × 10^−4^ Hz and ω_3_ = 7.324 × 10^−4^ Hz. G_i_(m) is the Galatry function which describes the direct absorption line shape for the H^18^OH (3662.9196 cm^−1^), H^16^OH (3663.0452 cm^−1^), H^16^OD (3663.2250 cm^−1^), H^17^OH (3663.3213 cm^−1^), H^16^OD (3663.3879 cm^−1^) and H^16^OD (3663.8419 cm^−1^) lines. The Galatry profile can be defined by:

(7) G(m)=AIln2γDπRe[∫0∞exp(−ixt−yt−zt−1+e−zt2z2)dt]

where $x=\sqrt{\ln2}\frac{\nu (m)-\nu_{0}}{\gamma_{D}}$, $y=\sqrt{\ln2}\frac{\gamma_{L}}{\gamma_{D}}$, $z=\sqrt{\ln2}\frac{\beta_{c}}{\gamma_{D}}$, *A_I_* the area under the line profile (cm^−1^), *ν*(*m*) the laser frequency (as a function of the acquired data point number *m*), *ν*_0_ (cm^−1^) is the line center frequency, *γ_D_* (cm^−1^) the Doppler half-width, *γ_L_* (cm^−1^) the collisional half-width, and *β_C_* (cm^−1^) representing the average effect of collisions on Doppler broadening.

To obtain the integrated absorbance *A_I_*, we fitted the F*_fit_* function (Equation (6)) to the experimentally recorded data with fixed isotope-dependent Doppler width *γ_D_* (scaled with the square root of the isotopologue mass), while the following parameters were determined in the fit: the integrated absorbance *A_I_*, the line center position *ν*_0_, the Lorentzian pressure broadening width *γ_L_*, the Dicke narrowing parameter *β_C_*, and the baseline modeling parameters.

**Figure 5.**
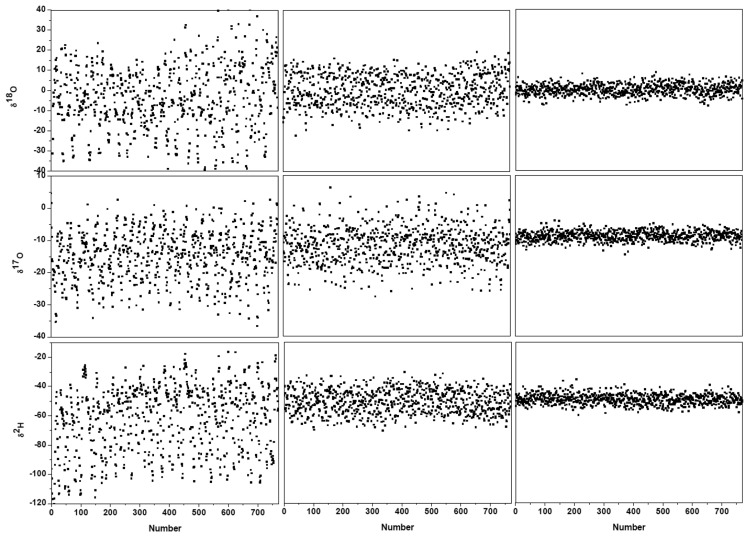
The ^18^O/^16^O, ^17^O/^16^O and D/H isotope ratios obtained with different baseline modelings. Left panel: baseline described with a 4th-order polynomial. Central panel: baseline modeled using a 4th-order polynomial and a Fourier series function (with three main frequency components). Right panel: no oscillation structure in original spectrum baseline.

Figure 3c and the black lines in Figure 4 show the results after suppression of the periodic undulation structure by fitting the spectrum to Equation (6). As can be observed in Figure 5, compared to the results obtained with a simple 4th-order polynomial as baseline model (left panel in Figure 5), baseline correction using a composition of a 4th-order polynomial and a Fourier series function (central panel in Figure 5) leads to a reduction of up to 3-fold in the standard deviation (SD) in the isotope ratio determination.

Though application of Fourier filtering can improve the measurement precision by removing oscillatory structure from the baseline, it is hard to completely account for the exact baseline structure with only three Fourier components. In fact, the residuals of Figure 3c still show signs of a residual periodic structure. We therefore proceeded to eliminate the source of the electrical perturbation, which was determined to be related to an electronic component failure in the preamplifier. In the case of no electrical perturbation, as shown in Figure 3d, a 3.7-fold improvement in the measurement precision was obtained (right panel in Figure 5), in comparison with the results achieved using a Fourier filtering (middle panel in Figure 5).

### 4.3. Allan Variance

The measurement precision is usually affected by the instrument instability and measurement errors related to sample handing and injection (e.g., incomplete evacuation of the gas cell between two consecutive measurements will lead to memory effects and thus affect the measurement precision). Another important limiting factor to achieving high precision is the signal-to-noise ratio (SNR) of the spectral data. In LAS, high SNR can be obtained by enhancing the signal (by selecting stronger absorption lines and using long absorption path length or cavity enhanced spectroscopy) and reducing the noise (by averaging N laser scans and using modulation or/and balanced-beam detection techniques). With the signal averaging approach, the optimal averaging number N, limited by the stability of the instrument, can be determined by an Allan variance analysis [36]. Alternatively, the individual spectral scans can be processed, and the resulting isotope ratios may be further averaged (within the system's stability time) to obtain the desired precision level. In the present work, due to laser wavelength drift, the maximum averaging number of the laser scans was limited at about N = 10, corresponding to an acquisition time of 1 s. The 1 s raw data were processed to provide 1 s raw δ values with a 1σ precision of 7.8‰ for δ^18^O, 6.6‰ for δ^17^O, and 8.0‰ for δ^2^H of a bottled water (Vittel, France).

**Figure 6.**
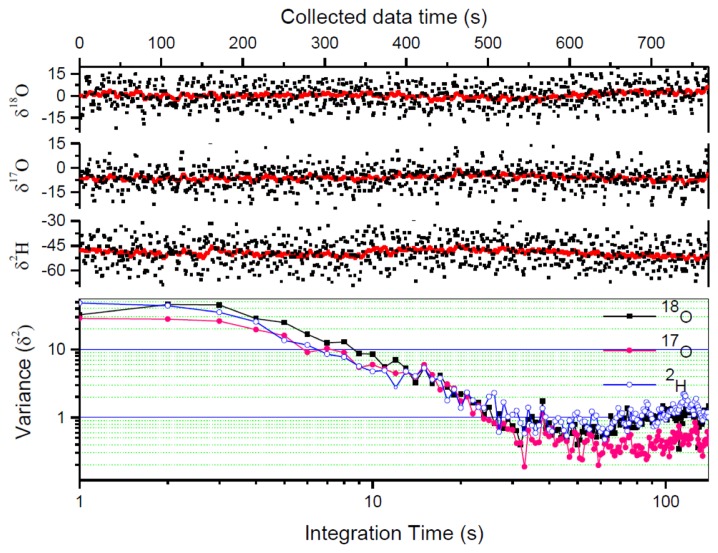
Measurement results of a bottled water (Vittel, France). The upper three panels show raw measurement of the δ-value for ^18^O, ^17^O, ^2^H (dots) and the corresponding Kalman filter output for a q-value of 800 (lines). The Allan variance plotted in the lower panel shows an optimal averaging time of ∼30 s for the present IRLS system.

In order to further improve the measurement precision, the 1 s raw δ values are further averaged. Allan variance analysis has been performed to determine the optimum averaging number. As can be seen in Figure 6 (lower panel), the optimum averaging time for the present instrument was ∼30 s. We then used conventional averaging of 30 measured δ -values corresponding to the optimum averaging time of 30 s. Herewith the precision was improved to 1.4‰ for δ^18^O, 1.1‰ for δ^17^O, and 1.5‰ for δ^2^H, respectively.

### 4.4. Kalman Filtering

Though signal averaging enables one to improve the measurement precision [1,11], such “post-processing” results in a slow temporal response of the system. For certain specific applications, such as the on-line monitoring of exhaled breath [37], it is highly desirable to be able to perform real-time measurements with high sensitivity and precision, while maintaining a fast system response. Kalman filtering, being an adaptive filtering technique uses a recursive procedure for “true value” prediction based on the previously determined values. It can efficiently remove the shot-to-shot variability related to the real-time noise in the measured data with minimal deformation of the physical quantity to be measured. Kalman filtering has been successfully applied before to real-time trace gas concentration measurements [38,39]. We recently introduced this technique to the field of isotope ratio measurements [28] in order to perform fast and high precision measurements. In this section, we describe in detail the theoretical consideration of a Kalman filter model for application to isotope ratio measurements.

Using a linear stochastic difference model, the true isotope ratio *δ̂_k_*_+1_ at time *k*+1 is evolved from the value *δ̂_k_* given at *k* according to:

(8) $${\hat{\delta }}_{k+1}={\hat{\delta }}_{k}+w_{k}$$

At time *k* the measured isotope ratio *z_k_* of the true value *δ̂_k_* can be expressed as follows:

(9) $$z_{k}={\hat{\delta }}_{k}+v_{k}$$

where *w*_k_ and *v*_k_ are uncorrelated random variables related to the process variability and the measurement noise with corresponding covariance of σ^2^*_w_* and σ^2^*_v_* respectively. Note that a hat above a variable indicates an estimated or predicted quantity, and a superscript negative sign above a variable indicates an *a priori* quantity. The recursive procedure involved in Kalman filtering may be considered to consist of two parts: the “time update” and the “measurement update”.

The time update procedure projects forward in time current isotope ratio and error variance estimates to obtain *a priori* estimates for the next time step. The prediction equations can be expressed as:

(10) $${\hat{\delta }}_{k+1}^{-}={\hat{\delta }}_{k}$$

(11) $$P_{k+1}^{-}=P_{k}+\sigma_{w}^{2}$$

The measurement update incorporates a new measurement into the *a priori* isotope ratio estimate to obtain an improved *a posteriori* estimate as filtered isotope ratio value, δ. The filtering equations involved in the measurement update are:

(12) $$K_{k}=P_{k}^{-}{\left(P_{k}^{-}+\sigma_{v}^{2}\right)}^{-1}$$

(13) $${\hat{\delta }}_{k}={\hat{\delta }}_{k}^{-}+K_{k}(z_{k}-{\hat{\delta }}_{k}^{-})$$

(14) $$P_{k}=P_{k}^{-}(1-K_{k})$$

where *δ̂*^−^*_k_*_+1_ is the predicted isotope ratio estimate at step k+1, *δ̂_k_* is the filtered isotope ratio value estimated at step k. *P*^−^*_k_*_+1_ is *a priori* estimate error variance, *P_k_* is *a posteriori* estimate error variance, *K_k_* is the Kalman gain that weights the measurement residual, defined as the difference between an actual isotope ratio measurement *z_k_* and a measurement prediction *δ̂*^−^*_k_* as shown in Equation (13).

In practical applications, the measurement noise σ^2^*_v_* and the true δ variability σ^2^*_w_* (related to real isotope abundance variation and real-time drifts resulting from laser frequency shift, thermal fluctuation, pressure variation, *etc.*) should be well defined.

Information on the measurement noise variance σ^2^*_v_* is usually known, because it depends on the quality of the measurement instrument, while the variance σ^2^*_w_* of the true isotopic ratio variability is quite subjective. Whereas both σ^2^*_v_* and σ^2^*_w_* vary with real variations of the isotope abundance, once the measurement system has reached equilibrium, the ratio of σ^2^*_v_* to σ^2^*_w_* should be constant. We therefore define:

(15) $$\text{q}={\sigma^{2}}_{v}/{\sigma^{2}}_{w}$$

**Figure 7.**
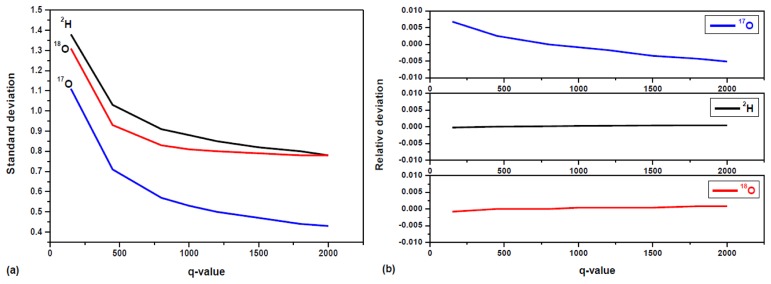
(**a**) Plots of the standard deviation in water (Vittel) isotope ratio determination in function of the q values involved in Kalman filtering; (**b**) The relative deviation in water (GS-42) isotope ratio measurement accuracy, (*δ* − *δ̄*);/*δ̄*, plotted in function of the q-parameter.

The parameter q is known as the filter tuning parameter. The choice of q depends on the particular instrument and its application environment. For a larger q value, the system's response time is longer to follow real-time variation in isotopic composition. Conversely, the filtering is less efficient in removing shot-to-shot real-time noise when a smaller q value is used. Figure 7a shows the 1σ precision (standard deviations) in isotope ratios determination of a bottled water (Vittel) as a function of q. As shown in Figure 7a, the improvement in the precision is clearly evident, as the standard deviation decreases exponentially with increased q value. The decreasing trend for δ^18^O is toward a steady state value for q > 800. For δ^17^O and δ^2^H, the decreasing trend slowed down for q > 800, while toward a steady state value for q > 2000. This may be explained by small drift in δ^17^O, δ^2^H resulting a Gaussian distribution, high q value is needed to get steady state, because the larger q value, the more efficient is the filtering δ-value fluctuations due to random noise. In the present work, a value of q = 800 was chosen as a compromise between fast temporal response and high filtering efficiency (thus high measurement precision) for the developed laser instrument. In the present work, a value of q = 800 was chosen as a compromise between fast temporal response and high filtering efficiency (thus high measurement precision) for the developed laser instrument. In our experiment, σ^2^*_v_* was determined by the δ-value variance deduced from the first 10 raw measurements, and σ^2^*_w_* was calculated by dividing σ^2^*_v_* by q.

The effects of the Kalman filtering on the measurement accuracy were investigated in the present work. For this purpose, the working standard GS-42 with well known isotope ratios was used. The relative deviation in water (GS-42) isotope ratio measurement accuracy, (*δ* − *δ̄*);/*δ̄*, is plotted in function of the q-parameter in Figure 7b. As can be seen, the relative deviation for δ^18^O and δ^2^H are almost constant in the range of ±1% with increased q-value. Though the relative deviation for δ^17^O varies evidently with q-value, the values are under a range of ±0.7%. The Kalman filtering is more effective to remove fluctuations due to random white noise. However, apart from the measurement random noise, temperature fluctuations of the gaseous sample and baseline drift may result in drift over time in δ values, which cannot be removed by the Kalman filtering. Because of slow drift in δ^17^O values, the fluctuation of δ^17^O mean for different q-value is almost two times larger than δ^18^O and δ^2^H. The maximal differences in relative deviation for the measurement accuracy are 1.6 × 10^−3^ for δ^18^O, 1 × 10^−2^ for δ^17^O, and 5 × 10^−4^ for δ^2^H in Figure 7b.

## 5. Results and Discussion

Table 2 summarizes the measurement accuracy of our IRLS instrument, compared to the IRMS calibrated value of GS-42.

The deviation of the measured values from the reference was −0.2‰ for δ^18^O, 1.4‰ for δ^17^O and 1.5‰ for δ^2^H. The large measurement deviation, especially for δ^17^O and δ^2^H, would be mainly caused sample mass effects. Though careful attention has been paid to sample amount effects in the present work, more precise control of injection volume and sample pressure was still needed. The 1σ standard deviation is 1.2‰ for δ^18^O, 0.7‰ for δ^17^O, and 1.4‰ for δ^2^H for five injections of water (GS-42). Moreover, the precision of 0.8‰ for δ^18^O, 0.6‰ for δ^17^O and 0.9‰ for δ^2^H with 1 s Kalman filtering also had certain influence on the measurement deviation. It should be noted that the δ^17^O value in water could not be directly determined with IRMS; the value given in the table is not a measured value, but was calculated based on a natural relation between the ^17^O and ^18^O abundance ratios [8]: δ^17^O = (1 + δ^18^O)^0.5281^ − 1.

The measurement precisions using 1 s Kalman filtering (q = 800) are given in parentheses in Table 2 in terms of the standard error of the mean values, SE (= SD/N^1/2^). We converted the standard deviation into SE with the same N values for the reason of comparison with the standard working reference GS-42.

Figure 6 shows raw measured results of a bottled water Vittel (black dots) in comparison with Kalman filtering results (black lines). With a q-value of 800, the Kalman filtered data obtained in 1 s reached a precision level better than the results obtained from conventional 30 s averaging. The 1σ standard deviation has been improved to 0.83‰ for δ^18^O, 0.57‰ for δ^17^O, and 0.91‰ for δ^2^H, with a measurement time of 1 s. Comparison of the measurement precisions is given in Table 3. 1 s Kalman filtering δ values (with q = 800) are compared with the raw measured δ values from the average of 10 laser scans (1 s) and the data from conventional averaging of 30 measured δ-value within the optimal averaging time of 30 s.

It is also worth noting, as can be seen in Figure 6, that the Kalman filtering does not affect the mean value of the data, and therefore does not affect the accuracy of the measurement.

## 6. Conclusions

We have described a compact IRLS instrument for simultaneous measurements of the D/H and ^18^O/^16^O, ^17^O/^16^O isotope ratios in water by laser absorption spectroscopy at 2.73 μm. We demonstrated the potential of application of Kalman filtering for fast and high precision isotope ratio measurements. The measurement precision achieved at 1 s Kalman filtering time intervals is better than that obtained by conventional 30 s averaging. The Kalman filter can be optimized to filter out the maximum amount of shot-to-shot real-time noise while following the real variation in measured physical quantity. The impact of the Kalman filtering on the measurement precision and accuracy were investigated. The measurement precision was improved clearly with high q value, while measurement accuracy was less impact by the Kalman filtering and mainly determined by calibration with known standard materials. The Voigt and Galatry profiles were used to investigate the possible influence of the choice of the line shape model on the determination of the integrated absorbance. The Galatry profile fit resulted in a smaller residual than using the Voigt profile. However, relatively big residuals around absorption peaks are still evidenced even using Galatry model, which leads to a bigger uncertainty in the determination of the integrated area A_I_. A more sophisticated model should be adopted in the future (for instance, the speed-dependent Galatry profile), to take into account simultaneously the narrowing due to the speed-dependence of relaxation rates and to the averaging effect of velocity-changing collisions. Fourier analysis of fitted spectral residuals allowed for determination of the Fourier frequency components in the undulation structure on the baseline. Reduction of up to three times in standard deviation in the isotope ratio determination was obtained by application of Fourier filtering to remove the undulation structure from spectrum baseline. However, a further reduction by a factor of 3.7 was obtained by the elimination of the electrical noise source. Measurement accuracy comparison of isotope ratios in water reference GS-42 between IRLS and IRMS was performed. Measured value of δ^18^O was more close to reference value, while measured values δ^17^O and δ^2^H presented a deviation from reference value. The measurement deviation would be mainly caused sample mass effects. Though careful attention has been paid to sample amount effects in the present work, more precise control of injection volume and sample pressure was still needed. Moreover, the precision of δ^18^O, δ^17^O and δ^2^H with 1 s Kalman filtering also had certain influence on the measurement deviation.

Further improvements in isotope ratio determination precision and accuracy can be envisaged as follows: (1) precise control of injection volume and sample pressure; (2) real-time calibration by alternating the introduction into the cell of a reference and the sample [21,40,41]; (3) real-time precise control and measurement of gas cell temperature for correction for temperature drifts during the measurements.

## Figures and Tables

**Table 1.** Comparisons of line intensities, ground state energies and temperature coefficients between different IRLS operating at different wavelengths in the infrared spectral region.

**Reference**	**Isotopologue**	**Frequency (cm^−1^)**	**Intensity (10^−23^ cm·mol^−1^)**	**Ground state energy (cm^−1^)**	**Temp. coef. at 296 K (K^−1^)**
	H^18^OH	7183.5858	0.62	733.7	6.9‰
[10,11]	H^16^OH	7183.6858	0.31	661.6	5.7‰
	H^17^OH	7183.7354	0.12	95	−3.4‰
	H^16^OD	7183.9727	0.035	156.4	−2.5‰
	H^18^OH	1483.9261	8.4	550.5	4.1‰
	H^16^OD	1484.1064	2.3	225.9	−1.3‰
[16]	H^16^OH	1484.2573	1.8	1899.0	26.2‰
	H^17^OH	1484.5109	2.0	205.5	−1.6‰
	H^18^OH	1484.9716	10.0	325.2	0.3‰
	H^16^OH	1485.1336	6.2	1907.6	26.4‰
	H^18^OH	3662.9196	2.1	398.3	1.5‰
[13,14] and *our work*	H^16^OH	3663.0452	8.5	586.4	4.6‰
	H^17^OH	3663.3213	7.2	224.3	−1.4‰
	H^16^OD	3663.8419	1.2	100.4	−3.4‰

**Table 2.** IRLS *vs.* IRMS: measurement accuracy comparison of isotope ratios in water reference GS-42. Where SE = SD/N^1/2^, is the standard error of the mean values for N measurements. IRLS results were obtained using 1 s Kalman filtering with q = 800.

	**N**	**δ^18^O (SE)**	**δ^17^O (SE)**	**δ^2^H (SE)**
IRMS (CIO)	3	−24.62 (0.02)	−13.1 (0.1)	−187.7 (0.3)
IRLS (present work)	5	−24.83 (0.50)	−11.7 (0.3)	−186.2 (0.6)

**Table 3.** Measurement precision comparison: Raw measured δ-value from the average of 10 laser scans in 1 s, data from conventional averaging of 30 measured δ-values within the optimal average time (30 s) determined by an Allan variance analysis, and 1 s Kalman filtering data with q = 800.

Method / Time	Measurement precision					
	δ^18^O (SD)		δ^17^O (SD)		δ^2^H (SD)	
Raw measurement / 1 s	7.8 ‰	6.6 ‰		8.0 ‰		
Kalman filtering / 1 s	1.4 ‰	1.1 ‰		1.5 ‰		
Averaging 30-δ / 30 s	0.8 ‰	0.6 ‰		0.9 ‰		
